# Serum 25-hydroxyvitamin D is associated with stroke history in a reverse J-shape

**DOI:** 10.3389/fneur.2022.1050788

**Published:** 2023-01-05

**Authors:** Jue-heng Pan, Shuo-long Wu, Jing-xiang Ma, Long Chang, Ying-feng Zheng, Xiao-dong Wang

**Affiliations:** ^1^Department of Neurosurgery, Affiliated Shunde Hospital, Ji'nan University, Foshan, China; ^2^Department of Neurosurgery, The First Affiliated Hospital, Ji'nan University, Guangzhou, China

**Keywords:** stroke, vitamin D, 25-hydroxyvitamin, stroke prevention, cross-sectional, NHANES

## Abstract

**Background:**

25-hydroxyvitamin D [25(OH)D], the major form of vitamin D in the body, has a non-linear association with stroke risk. However, the association is not fully understood. The specific shape of the association and the ideal value of 25(OH)D related to minimum risk of stroke remain unclear.

**Aim:**

We conducted the study to establish the correlation between circulating 25(OH)D and stroke history and determine the ideal value of 25(OH)D in relation to the lowest stroke prevalence.

**Methods:**

Data from the National Health and Nutrition Examination Survey (NHANES) were used for analyzes. We used multivariate logistic regression analysis with fitted smooth curves to explore the relationship between 25(OH)D and self-reported stroke history. Subsequently, 40,632 participants were enrolled in the study.

**Results:**

A reverse J-shaped association between 25(OH)D and stroke history was determined, where the lowest stroke prevalence for the 25(OH)D level was about 60 nmol/L. After adjusting for confounding factors, prevalence of stroke showed an increasing trend below and above the middle quintile (53.2–65.4 nmol/L) of 25(OH)D. Participants with 25(OH)D levels in the lowest quintile (≤ 39.3 nmol/L) had a 38% increased prevalence of stroke (OR 1.38, 95 %CI 1.12–1.70), while those in the higher level range of 25(OH)D (65.5–80.8 nmol/L) had a 27% higher stroke prevalence (OR 1.27, 95 %CI 1.03–1.57).

**Conclusion:**

Using data from a large, cross-sectional cohort program, we found that circulating 25(OH)D was related to stroke history in a reverse J-shaped manner. Given how the causal relationship between circulating 25(OH)D and history of stroke has not been established, more high-quality evidence based on the reverse J-shaped feature is needed to elucidate the link between vitamin D and stroke risk, and the effect of vitamin D supplements on stroke prevention.

## 1. Introduction

Stroke is estimated to be the third leading cause of both death and disability throughout the world (1). Between 1990 and 2019, the prevalence of stroke among people under the age of 70 increased by 22.0% and the incidence rate increased by 15.0% (1). Primary prevention strategies have focused on the modifiable risk factors for stroke, including elevated systolic blood pressure, body-mass index and fasting plasma glucose, ambient particulate matter pollution, and smoking (1). Recently, 25-hydroxyvitamin D 25(OH)D, the main form of vitamin D in the body, is considered to be a potential modifiable risk factor of stroke (2–5). Low 25(OH)D level is significantly related to higher risk of stroke (2, 3, 5, 6). However, studies of vitamin D supplementation have failed to show benefits in reducing stroke risk (4, 7, 8). One possible reason for this is that 25(OH)D concentrations are not linearly related to stroke risk (3, 9, 10). Few articles have examined the specific shape of the non-linear relationship and the ideal value of 25(OH)D related to the minimized risk of stroke. However, the relatively small sample size and limited data on serum 25(OH)D limit the analysis of the association with stroke risk at serum 25(OH)D levels >110 nmol/L (9, 10). A meta-analysis reported that the incidence of stroke was minimized at 50 nmol/L of 25(OH)D (3). But the use of average or median amount of 25(OH)D concentration for analysis may have missed the impact of specific data on the results. Thus, we conducted this study based on a large-scale, cross-sectional project from the National Health and Nutrition Examination Survey (NHANES) (2001–2018) to establish the correlation between them and calculate the optimal value of 25(OH)D in the United States.

## 2. Methods

### 2.1. Data sources

This study was based on data from NHANES. NHANES is a program approved by the National Center for Health Statistics (NCHS) Research Ethics Review Board. About 10,000 people take part in each of the program's 2-year cycles.

### 2.2. Participants

**Figure 1:**
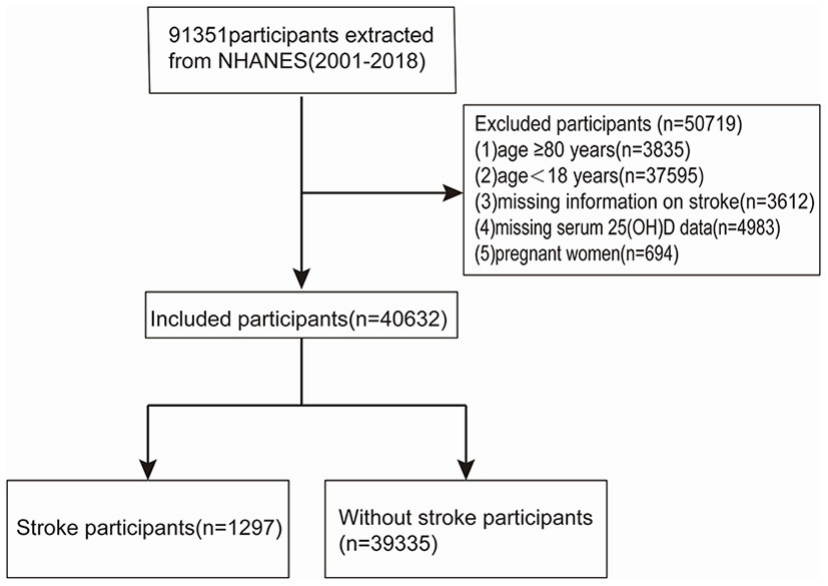
Flow chart of study population inclusion.

A total of 91,351 participants were recruited from 2001 to 2018, representing nine NHANES cycles. Individuals 80 years old and older were top-coded at 80 years of age since 2007, thus, we excluded participants with an age ≥80 years (*n* = 3,835), <18 years (*n* = 37,595), missing information on stroke (*n* = 3,612), missing circulating 25(OH)D data (*n* = 4,983), and pregnant women (*n* = 694). Subsequently, 40,632 participants in all were enrolled (Figure 1).

### 2.3. Serum 25(OH)D concentration assessment

Serum specimens were collected from participants in a specially equipped and designed Mobile Examination Center (MEC). Detailed instructions on specimen collection and processing are discussed in the NHANES Laboratory Procedures Manual (https://wwwn.cdc.gov/nchs/data/nhanes/2017-2018/manuals/2017_MEC_Laboratory_Procedures_Manual.pdf). Serum 25(OH)D concentration from NHANES 2001 to 2006 were measured by the Diasorin radioimmunoassay (RIA) kit (Stillwater, MN, USA). By using regression, the data were converted into equivalent 25(OH)D measurements from an established liquid chromatography-tandem mass spectrometry (LC-MS/MS) technique, which was used in the analysis of circulating 25(OH)D data since 2007. The NCHS of the Centers for Disease Control and Prevention (CDC) recommend using the LC-MS/MS-equivalent data for all analyzes across different survey cycles of the NHANES. The detailed note is available online (https://wwwn.cdc.gov/nchs/nhanes/vitamind/analyticalnote.aspx).

### 2.4. Stroke history assessment

Stroke history was identified through self-reports. The participants with a “yes” to the question “Has a doctor or other health professional ever told you that you had a stroke?” were considered to have stroke history. The mean (± standard deviation [SD]) interval between stroke and measurement of serum 25(OH)D concentration was 8.95 ± 9.37 years, the median was 6 years, with a range of 0–69 years.

### 2.5. Variables

According to the literature (3, 9, 11), the basal features of the individuals were gathered, including age, gender, ethnicity (Mexican Americans, other Hispanics, non-Hispanic white, non-Hispanic black, other races), marital status (we defined married/living with partner as “living with partner,” widowed/divorced/unmarried as “living without partner”), education level (“less than high school,” “high school,” and “college or above”), season of examination (“November 1 through April 30” was defined as “winter months” and “May 1 through October 31” as “summer months”), body mass index (BMI), cotinine (a biomarker of tobacco use), urine creatinine, total cholesterol (TC), and high density lipoprotein cholesterol (HDLC). The history of hypertension, hypercholesterolemia, diabetes, asthma, emphysema, chronic bronchitis, and cardiac disease (congestive heart failure/coronary heart disease/angina/heart attack) were assessed from the Medical Conditions Questionnaire (MCQ) by self-reporting.

### 2.6. Statistical analysis

The outliers in continuous variables did not significantly alter the mean and were therefore included. Missing data in categorical variables were defined as “Missing.” For the missing values of the continuous variables, no changes were made, as <5% of the data were missing (Supplementary Table S1).

Baseline characteristics between groups were compared using *t*-test (2-tailed) for continuous variables and Pearson's chi-square test for categorical variables. Continuous variables were presented as means ± standard deviation (SD) and categorical variables as frequency and percentage (*n*, %). Univariate and multivariate logistic regression analysis were applied to explore the link between circulating 25(OH)D level and history of stroke. We performed three models—crude model without adjusted covariates; model I with adjusted gender, age and ethnicity; and model II with complete adjustment. The covariates were adjusted in model II when added to this model, matched odds ratio changed by more than 10%.

To identify the non-linear correlation between concentration of 25(OH)D and stroke history, fitted smooth curves were plotted using generalized additive model with full adjustment. If a non-linear association was observed, piece-wise linear regression model was performed to determine the inflection point by logarithm likelihood ratio test using two steps recursive method.

Stratified and interaction analyzes were conducted according to age (“ ≤ 40,” “>40, ≤ 60,” and “>60” years), BMI (“ <25,” “≥25, <30,” and “≥30” kg/m^2^), HDLC (quartile: “0.16–1.03,” “1.06–1.27,” “1.29–1.55,” “1.58–5.84” mmol/L), gender, ethnicity, marital status, education level, hypertension, hypercholesterolemia, diabetes, asthma, emphysema, chronic bronchitis, and cardiac disease by multivariate logistic regression with full adjustment. For the sensitivity analysis, we repeated the main analysis using five imputed data sets generated by multiple imputation based on the method of chained equation approximation. Statistical analysis and graphics were carried out with the software package R-3.4.3 (https://www.R-project.org; the R Foundation, Vienna, Austria) and Empower-Stats (https://www.empowerstats.com, X&Y Solutions, Inc., Boston, MA, USA). The *P* value of < 0.05 was considered statistically significant.

## 3. Results

In Table 1, the baseline features of the participants were presented. There were 1,297 participants (632 men and 665 women) with stroke history included in this study. Individuals with a history of stroke were more likely to be older, non-Hispanic white and black, less educated, and living alone. Moreover, they had higher BMI and cotinine, but low total cholesterol (TC), high density lipoprotein cholesterol (HDLC) and urine creatinine levels. Additionally, they had more traditional risk factors such as hypertension, diabetes, hypercholesterolemia, and a higher prevalence of asthma, emphysema, chronic bronchitis and cardiac disease. Most participants (95.66%) had a level of 25(OH)D ≤ 110 nmol/L. Compared to the middle quintile (53.2–65.4 nmol/L) of 25(OH)D, the percentage of subjects with stroke was higher in the first (≤ 39.3 nmol/L) and the fifth quintile (≥80.9 nmol/L), 23.05% vs. 14.80% and 24.83% vs. 14.80%, respectively.

**Table 1:** Baseline characteristics of participants with complete data (no imputation).

**Variables**	**Total** **(*n* = 40,632)**	**Stroke ** **(*n* = 1,297)**	**Without stroke ** **(*n* = 39,335)**	***P*-value**
Age (years, mean ± sd)	47.67 ± 16.49	62.41 ± 12.22	47.18 ± 16.39	<0.001
BMI (kg/m^2^, mean ± sd)	29.18 ± 6.93	30.54 ± 7.01	29.14 ± 6.92	<0.001
Cotinine (ng/ml, mean ± sd)	60.09 ± 129.28	84.29 ± 150.82	59.29 ± 128.43	<0.001
TC (mmol/L, mean ± sd)	5.04 ± 1.10	4.88 ± 1.22	5.05 ± 1.09	<0.001
HDLC (mmol/L, mean ± sd)	1.36 ± 0.41	1.33 ± 0.42	1.37 ± 0.41	<0.001
Urine creatinine (μmol/L, mean ± sd)	11,240.07 ± 7,229.44	10,747.20 ± 7,101.87	11,255.62 ± 7,232.97	0.015
**Gender (** ***n*** **, %)**				
Male	19,967 (49.14%)	632 (48.73%)	19,335 (49.15%)	0.764
Female	20,665 (50.86%)	665 (51.27%)	20,000 (50.85%)	
**Season of examination (** ***n*** **, %)**				
Winter months	19,331 (47.58%)	621 (47.88%)	18,710 (47.57%)	0.824
Summer months	21,301 (52.42%)	676 (52.12%)	20,625 (52.43%)	
**Ethnicity (** ***n*** **, %)**				
Mexican American	7,097 (17.47%)	163 (12.57%)	6,934 (17.63%)	<0.001
Other Hispanic	3,564 (8.77%)	75 (5.78%)	3,489 (8.87%)	
Non-Hispanic white	17,175 (42.27%)	572 (44.10%)	16,603 (42.21%)	
Non-Hispanic black	8,636 (21.25%)	389 (30.07%)	8,246 (20.96%)	
Other race	4,160 (10.24%)	97 (7.48%)	40,643 (10.33%)	
**Education level (** ***n*** **, %)**				
Less than high school	10,244 (25.21%)	470 (36.24%)	9,774 (24.85%)	<0.001
High school	9,372 (23.07%)	332 (25.60%)	9,040 (22.98%)	
College or above	20,983 (51.64%)	494 (38.09%)	20,489 (52.09%)	
**Marital status (** ***n*** **, %)**				
Living with partner	24,954 (61.41%)	708 (54.59%)	24,246 (61.64%)	<0.001
Living without partner	15,658 (38.54%)	588 (45.34%)	15,070 (38.32%)	
**Hypertension (** ***n*** **, %)**				
Yes	13,613 (33.50%)	985 (75.94%)	12,628 (32.10%)	<0.001
No	26,885 (66.17%)	309 (23.82%)	26,576 (67.56%)	
**Hypercholesterolemia (** ***n*** **, %)**				
Yes	12,763 (31.41%)	725 (55.90%)	12,038 (30.60%)	<0.001
No	20,873 (51.37%)	476 (36.7%)	20,397 (51.85%)	
**Diabetes (** ***n*** **, %)**				
Yes	4,910 (12.08%)	446 (34.39%)	4,464 (11.35%)	<0.001
No	35,695 (87.85%)	848 (65.38%)	34,847 (88.59%)	
**Asthma (** ***n*** **, %)**				
Yes	5,627 (13.85%)	291 (22.44%)	5,336 (13.57%)	<0.001
No	34,969 (86.06%)	1,005 (77.49%)	33,964 (86.35%)	
**Emphysema (** ***n*** **, %)**				
Yes	755 (1.86%)	97 (7.48%)	658 (1.67%)	<0.001
No	39,836 (98.04%)	1,196 (92.21%)	38,640 (98.23%)	
**Chronic bronchitis (** ***n*** **, %)**				
Yes	2,334 (5.74%)	167 (12.88%)	2,167 (5.51%)	<0.001
No	38,216 (94.05%)	1,125 (86.74%)	37,091 (94.30%)	
**Cardiac disease (** ***n*** **, %)**				
Yes	3,003 (7.39%)	477 (36.78%)	2,526 (6.42%)	<0.001
No	37,629 (92.61%)	820 (63.22%)	36,809 (93.58%)	
25(OH)D (nmol/L, mean ± sd)	61.74± 26.08	63.24 ± 29.81	61.69 ± 25.95	0.035
**25(OH)D (** ***n*** **, %)**				
≤ 110 nmol/L	38,868 (95.66%)	1,213 (93.52%)	37,655 (95.73%)	<0.001
>110 nmol/L	1,764 (4.34%)	84 (6.48%)	1,680 (4.27%)	
**25(OH)D quintile [nmol/L**, ***n*** **(%)]**				
Q1 (≤ 39.3)	8,097 (19.93%)	299 (23.05%)	7,798 (19.82%)	<0.001
Q2 (39.4–53.1)	8,071 (19.86%)	244 (18.81%)	7,827 (19.90%)	
Q3 (53.2–65.4)	8,113 (19.97%)	192 (14.80%)	7,921 (20.14%)	
Q4 (65.5–80.8)	8,196 (20.17%)	240 (18.50%)	7,956 (20.23%)	
Q5 (≥80.9)	8,155 (20.07%)	322 (24.83%)	7,833 (19.91%)	

The percentage may not add up to 100% due to rounding or missing values.

BMI, body mass index; TC, total cholesterol; HDLC, high density lipoprotein cholesterol; 25(OH)D, 25-hydroxyvitamin D.

Age, ethnicity, education level, marital status, BMI, cotinine, TC, HDLC, urine creatinine and history of hypertension, diabetes, hypercholesterolemia, asthma, emphysema, chronic bronchitis, cardiac disease (*P* values < 0.05) were considered as potential risk factors for stroke history (Table 2). After fully adjusting for potential factors in Model II (Table 3), when taking the third quintile (53.2–65.4 nmol/L) of 25(OH)D as reference, stroke prevalence showed an increasing trend below and above the reference range, with a 38% increased prevalence of stroke (OR 1.38, 95 %CI 1.12–1.70) in the lowest level group (≤ 39.3 nmol/L) and 27% (OR 1.27, 95 %CI 1.03–1.57) in the fourth quintile (65.5–80.8 nmol/L). Stroke prevalence in the highest level group (≥80.9 nmol/L) of 25(OH)D was slightly lower than that in the first quintile (OR 1.36, 95 %CI 1.11–1.66).

**Table 2:** Univariate analysis for stroke history with complete data.

	**Statistics**	**OR (95 %CI)**	***P*-value**
Age (years, mean ± sd)	47.67 ± 16.49	1.07 (1.06, 1.07)	<0.001
**Gender (** ***n*** **, %)**			
Male	19,967 (49.14%)	Ref	
Female	20,665 (50.86%)	1.02 (0.91, 1.14)	0.762
**Ethnicity (** ***n*** **, %)**			
Mexican American	7,097 (17.47%)	Ref	
Other Hispanic	3,564 (8.77%)	0.91 (0.69, 1.21)	0.526
Non-Hispanic white	17,175 (42.27%)	1.47 (1.23, 1.75)	<0.001
Non-Hispanic black	8,636 (21.25%)	2.01 (1.67, 2.42)	<0.001
Other race	4,160 (10.24%)	1.02 (0.79, 1.31)	0.905
**Education level (** ***n*** **,%)**			
Less than high school	10,244 (25.21%)	Ref	
High school	9,372 (23.07%)	0.76 (0.66, 0.88)	<0.001
College or above	20,983 (51.64%)	0.50 (0.44, 0.57)	<0.001
Missing	33 (0.08%)	0.65 (0.09, 4.77)	0.672
**Marital status (** ***n*** **,%)**			
Living with partner	24,954 (61.41%)	Ref	
Living without partner	15,658 (38.54%)	1.34 (1.20, 1.49)	<0.001
Missing	20 (0.05%)	1.80 (0.24, 13.48)	0.566
BMI (kg/m^2^, mean ± sd)	29.18 ± 6.93	1.03 (1.02, 1.03)	<0.001
Cotinine (ng/ml, mean ± sd)	60.09 ± 129.28	1.00 (1.00, 1.00)	<0.001
TC (mmol/L, mean ± sd)	5.04 ± 1.10	0.86 (0.82, 0.91)	<0.001
HDLC (mmol/L, mean ± sd)	1.36 ± 0.41	0.79 (0.68, 0.91)	<0.001
Urine creatinine (μmol/L, mean ± sd)	11,240.07± 7,229.44	1.00 (1.00, 1.00)	0.015
**Hypertension (** ***n*** **, %)**			
Yes	13,613 (33.50%)	Ref	
No	26,885 (66.17%)	0.15 (0.13, 0.17)	<0.001
Missing	134 (0.33%)	0.29 (0.09, 0.92)	0.036
**Hypercholesterolemia (** ***n*** **,%)**			
Yes	12,763 (31.41%)	Ref	
No	20,873 (51.37%)	0.39 (0.34, 0.44)	<0.001
Missing	6,996 (17.22%)	0.23 (0.19, 0.29)	<0.001
**Diabetes (** ***n*** **, %)**			
Yes	4,910 (12.08%)	Ref	
No	35,695 (87.85%)	0.24 (0.22, 0.27)	<0.001
Missing	27 (0.07%)	1.25 (0.38, 4.17)	0.715
**Asthma (** ***n*** **, %)**			
Yes	5,627 (13.85%)	Ref	
No	34,969 (86.06%)	0.54 (0.47, 0.62)	<0.001
Missing	36 (0.09%)	0.52 (0.07, 3.84)	0.525
**Emphysema (** ***n*** **, %)**			
Yes	755 (1.86%)	Ref	
No	39,840 (98.04%)	0.21 (0.17, 0.26)	<0.001
Missing	41 (0.10%)	0.73 (0.26, 2.10)	0.564
**Chronic bronchitis (** ***n*** **, %)**			
Yes	2,334 (5.74%)	Ref	
No	38,216 (94.05%)	0.39 (0.33, 0.47)	<0.001
Missing	82 (0.20%)	0.84 (0.34, 2.11)	0.715
**Cardiac disease (** ***n*** **, %)**			
Yes	3,003 (7.39%)	Ref	
No	37,629 (92.61%)	0.12 (0.10, 0.13)	<0.001
**Season of examination (** ***n*** **, %)**			
Winter months	19,331 (47.58%)	Ref	
Summer months	21,301 (52.42%)	0.99 (0.88, 1.10)	0.824
25(OH)D (nmol/L)	61.74 ± 26.08	1.00 (1.00, 1.00)	0.035
**25(OH)D quartile (nmol/L)**			
Q1 (≤ 39.3)	8,097 (19.93%)	Ref	
Q2 (39.4–53.1)	8,071 (19.86%)	0.81 (0.68, 0.97)	0.018
Q3 (53.2–65.4)	8,113 (19.97%)	0.63 (0.53, 0.76)	<0.001
Q4 (65.5–80.8)	8,196 (20.17%)	0.79 (0.66, 0.94)	0.007
Q5 (≥80.9)	8,155 (20.07%)	1.07 (0.91, 1.26)	0.395

The percentage may not add up to 100% due to rounding or missing values.

BMI, body mass index; TC, total cholesterol; HDLC, high density lipoprotein cholesterol; 25(OH)D, 25-hydroxyvitamin D; OR, odds ratio; CI, confidence interval; Ref, reference.

**Table 3:** Relationship between 25(OH)D and stroke history in different models with complete data.

**Variable**	***n***	**Stroke (*n*, %)**	**Crude model**	**Model I**	**Model II**
			**OR (95 %CI)**	**OR (95 %CI)**	**OR (95 %CI)**
25(OH)D	40,632	1,297 (3.19)	1.00 (1.00, 1.00)^*^	1.00 (1.00, 1.00)^*^	1.00 (1.00, 1.00)
**25(OH)D quintile (nmol/L)**				
Q1 (≤ 39.3)	8,097	299 (3.69)	1.58 (1.32, 1.90)^**^	1.63 (1.34, 1.97)^**^	1.38 (1.12, 1.70)^**^
Q2 (39.4–53.1)	8,071	244 (3.02)	1.29 (1.06, 1.56)^*^	1.35 (1.11, 1.64)^**^	1.32 (1.07, 1.63)^*^
Q3 (53.2–65.4)	8,113	192 (2.37)	Ref	Ref	Ref
Q4 (65.5–80.8)	8,196	240 (2.93)	1.24 (1.03, 1.51)^*^	1.13 (0.93, 1.38)	1.27 (1.03, 1.57)^*^
Q5 (≥80.9)	8,155	322 (3.95)	1.70 (1.41, 2.03)^**^	1.24 (1.03, 1.49)^*^	1.36 (1.11, 1.66)^**^

Crude model: no adjusted. Model I: adjusted for age, gender, ethnicity. Model II: adjusted for age, gender, ethnicity, marital status, education level, season of examination, BMI, cotinine, urine creatinine, TC, HDLC, hypertension, hypercholesterolemia, diabetes, asthma, emphysema, cardiac disease.

BMI, body mass index; TC, total cholesterol; HDLC, high density lipoprotein cholesterol; 25(OH)D, 25-hydroxyvitamin D; OR, odds ratio; CI, confidence interval.

^*^P < 0.05.

^**^P < 0.01.

**Figure 2:**
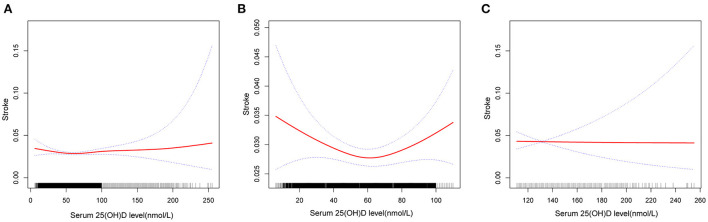
Association between serum 25(OH)D level and history of stroke. **(A)** Reverse J-shape association between serum 25(OH)D level and history of stroke in the study population (*n* = 40,632). **(B)** Association between them in the population with serum 25(OH)D level ≤ 110 nmol/L (*n* = 38,868). **(C)** Association between them in the population with serum 25(OH)D level >110 nmol/L (*n* = 1,764). Solid red line represents the smooth curve fit between variables. Blue bands represent the 95% of confidence intervals. All adjusted for age, gender, ethnicity, marital status, education level, season of examination, BMI, cotinine, urine creatinine, TC, HDLC, hypertension, hypercholesterolemia, diabetes, asthma, emphysema, cardiac disease.

Fitted smooth curves identified that circulating 25(OH)D had a reverse J-shaped association with stroke history (Figure 2). A significant point with the lowest stroke prevalence was observed in the population with serum 25(OH)D level ≤ 110 nmol/L. In the case of serum 25(OH)D below 58.7 nmol/L (Table 4), increased circulating 25(OH)D was related to decreased prevalence of stroke (OR 0.99, 95 %CI 0.99–1.00). When the 25(OH)D level was higher than 58.7 nmol/L, the stroke prevalence elevated with the increase of 25(OH)D concentration, until 67.7 nmol/L (OR 1.14, 95 %CI 1.03–1.26). 25(OH)D concentrations above 67.7 nmol/L were not significantly correlated with stroke history (OR 1.00, 95 %CI 1.00–1.00).

**Table 4:** Inflection point analysis of serum 25(OH)D based on piece-wise linear regression model.

		**Adjusted OR (95 %CI), *P*-value**
Point 1	Fitting by the standard linear model	1.00 (1.00, 1.00) 0.241
	Fitting by the piece-wise linear model	
	Inflection point	58.7
	serum 25(OH)D <58.7 (nmol/L)	0.99 (0.99, 1.00) 0.041
	serum 25(OH)D >58.7 (nmol/L)	1.00 (1.00, 1.01) 0.061
	Log likelihood ratio	0.021
Point 2	Fitting by the standard linear model	1.00 (1.00, 1.01) 0.246
	Fitting by the piece-wise linear model	
	Inflection point	67.7
	serum 25(OH)D >58.7, <67.7 (nmol/L)	1.05 (1.01, 1.10) 0.022
	serum 25(OH)D >67.7 (nmol/L)	1.00 (1.00, 1.00) 0.657
	Log likelihood ratio	0.027

Adjusted for age, gender, ethnicity, marital status, education level, season of examination, BMI, cotinine, urine creatinine, TC, HDLC, hypertension, hypercholesterolemia, diabetes, asthma, emphysema, cardiac disease.

BMI, body mass index; TC, total cholesterol; HDLC, high density lipoprotein cholesterol; 25(OH)D, 25-hydroxyvitamin D; OR, odds ratio; CI, confidence interval.

Subgroup analysis showed similar relationship in most subgroups accounting for additional confounding factors: the middle group (53.2–65.4 nmol/L) of 25(OH)D had the minimized stroke prevalence (Table 5 and Supplementary Table S3). The relationship was weaker among participants with emphysema and cardiac disease, and individuals with BMI <25 kg/m^2^, HDLC <1.04 mmol/L. However, interaction analyzes did not reveal any statistical interactions between all variables and serum 25(OH)D. The results were consistent across analyzes of the complete case data and the imputed datasets (Supplementary Table S2).

**Table 5:** Adjusted odds ratio for the association of 25(OH)D and stroke history in subgroups by age, emphysema, cardiac disease, BMI, and HDLC.

	***n***	**25(OH)D Q1**	**25(OH)D Q2**	**25(OH)D Q3**	**25(OH)D Q4**	**25(OH)D Q5**	***P* for interaction**
		**OR (95 %CI)**	**OR (95 %CI)**	**OR (95 %CI)**	**OR (95 %CI)**	**OR (95 %CI)**	
**Age (years)**							
≤ 40	15,215	1.32 (0.58, 3.00)	1.73 (0.82, 3.66)	Ref	1.28 (0.56, 2.93)	1.27 (0.53, 3.06)	0.895
>40, ≤ 60	14,379	1.26 (0.88, 1.80)	1.10 (0.75, 1.59)	Ref	1.19 (0.82, 1.74)	1.44 (0.99, 2.08)	
>60	11,038	1.48 (1.12, 1.94)^*^	1.44 (1.09, 1.89)^*^	Ref	1.33 (1.02, 1.73)^*^	1.38 (1.08, 1.78)^*^	
**Emphysema**							
Yes	755	1.00 (0.46, 2.18)	1.07 (0.48, 2.36)	Ref	0.65 (0.27, 1.55)	1.13 (0.55, 2.31)	0.228
No	39,836	1.54 (1.23, 1.93)^*^	1.34 (1.08, 1.68)^*^	Ref	1.33 (1.07, 1.65)^*^	1.38 (1.12, 1.70)^*^	
**Cardiac disease**							
Yes	3,003	1.06 (0.75, 1.50)	1.18 (0.83, 1.67)	Ref	1.08 (0.76, 1.53)	1.18 (0.84, 1.65)	0.324
No	37,629	1.60 (1.23, 2.09)^*^	1.40 (1.07, 1.82)^*^	Ref	1.42 (1.09, 1.85)^*^	1.47 (1.14, 1.89)^*^	
**BMI (kg/m** ^2^ **)**							
<25	11,515	1.09 (0.67, 1.77)	0.97 (0.59, 1.59)	Ref	1.17 (0.74, 1.85)	1.34 (0.88, 2.05)	0.459
≥25, <30	13,349	1.53 (1.04, 2.24)^*^	1.47 (1.01, 2.13)^*^		1.06 (0.72, 1.55)	1.38 (0.97, 1.97)	
≥30	15,182	1.40 (1.04, 1.89)^*^	1.35 (0.99, 1.82)	Ref	1.50 (1.11, 2.03)^*^	1.33 (0.98, 1.81)	
**HDLC, quartile (mmol/L)**							
Q1 (0.16–1.03)	8,970	1.07 (0.72, 1.59)	1.13 (0.78, 1.66)	Ref	1.16 (0.79, 1.71)	1.21 (0.82, 1.78)	0.389
Q2 (1.06–1.27)	10,537	1.44 (0.96, 2.15)	1.30 (0.88, 1.94)	Ref	1.02 (0.67, 1.54)	1.54 (1.05, 2.25)^*^	
Q3 (1.29–1.55)	10,217	1.84 (1.16, 2.93)^*^	2.03 (1.28, 3.23)^*^	Ref	1.63 (1.03, 2.60)^*^	1.36 (0.87, 2.15)	
Q4 (1.58–5.84)	10,715	1.36 (0.87, 2.14)	1.06 (0.65, 1.74)	Ref	1.40 (0.90, 2.18)	1.30 (0.85, 1.99)	

Adjusted for age, gender, ethnicity, marital status, education level, season of examination, BMI, cotinine, urine creatinine, TC, HDLC, hypertension, hypercholesterolemia, diabetes, asthma, emphysema and cardiac disease except the subgroup variable.

BMI, body mass index; TC, total cholesterol; HDLC, high density lipoprotein cholesterol; 25(OH)D, 25-hydroxyvitamin D; OR, odds ratio; CI, confidence interval; Ref, reference.

^*^P < 0.05.

## 4. Discussion

In this cross-sectional study of 40,632 participants in the US, we indicated that circulating 25(OH)D was in related to stroke history in a reverse J-shaped manner, with the optimal 25(OH)D value at about 60 nmol/L.

The non-linear shape demonstrated in this study was similar to a cohort study of 3,458 participants with concentrations of 25(OH)D <110 nmol/L from Hong Kong (9), in which the optimal value was observed between 70 and 80 nmol/L. Furthermore, we showed that increased level of 25(OH)D >110 nmol/L was not related with increase in stroke prevalence. Similarly, this pattern was consistent with that of relation between 25(OH)D and risk of recurrent stroke identified in a recent research based on the data from United Kingdom Biobank (12). These studies from different populations confirmed the non-linear correlation between 25(OH)D and stroke risk with or without a history of stroke.

Notably, serum 25(OH)D level related to the lowest stroke prevalence was about 60 nmol/L, similar to the optimal level with regard to recurrent stroke (12). It was higher than that in a study which reported the incidence of stroke was minimized at 50 nmol/L of 25(OH)D level (3). Their use of the average or median 25(OH)D level for analysis in that study may have missed the impact of specific data on the results. Besides, we found the saturation value at 67.7 nmol/L above which circulating 25(OH)D was not correlated with stroke history. These results revealed that circulating 25(OH)D may serve different roles at different concentrations. Low concentrations, especially when <30 nmol/L of 25(OH)D are related to higher incidence (5, 6, 9) and recurrence (12–15) of stroke. Vitamin D supplements may help reduce stroke risk when 25(OH)D levels are lower than the optimal value but may have deleterious or no effects when 25(OH)D exceed the optimal point.

Some vitamin D supplementation studies have been conducted, but most of the results were disappointing (4, 7, 8, 16, 17). In a nationwide randomized, placebo-controlled trial with a median follow-up of 5.3 years, supplements with vitamin D were not related to reduced risk of stroke (RR 0.95, 95 %CI 0.76–1.20) (17). Recent research indicated that compared to placebo, supplementing with vitamin D was not related to reduced stroke (RR 1.06, 95 %CI 0.98–1.15) (8). Based on our findings, two points should be noted regarding vitamin D supplementation: firstly, participants with basal concentration of circulating 25(OH)D above the optimal point, ~60 nmol/L should be excluded, as supplementation of vitamin D would not benefit and can rather increase stroke risk. The average level of circulating 25(OH)D at baseline in the nationwide randomized, placebo-controlled trial was 77 nmol/L, with only 12.7% participants with levels below 50 nmol/L (17). Recently, research found that supplements with vitamin D3 did not reduce the rate of major cardiovascular events, probably due to the fact that most participants had sufficient vitamin D at baseline (75 ± 18 nmol/L) (7). Similarly, vitamin D supplementation significantly improved stroke outcome in participants with basal circulating 25(OH)D levels ≤ 20 ng/ml (50 nmol/L), but not in those with basal concentrations of 21–29 ng/ml (52.5–72.5 nmol/L) (18). Secondly, the circulating 25(OH)D concentration must be carefully monitored after vitamin D supplementation. Due to individual differences in response to drugs and genetic polymorphisms in vitamin D-binding proteins (19, 20), circulating 25(OH)D may remain deficient or may even rise to harmful levels in some subjects after vitamin D supplementation, thus affecting the results. Besides, it will enable us to further understand at what level and to what extent circulating 25(OH)D affects the incidence of stroke.

The strength of this study is the inclusion of a large, population-based data that provides strong evidence to establish the correlation between 25(OH)D and stroke history. In addition, to the best of our knowledge, this is the first paper to utilize the US national database for stroke to identify the optimal value of serum 25(OH)D. However, several limitations are noted. First, the single-measurement of circulating 25(OH)D concentration and the other covariates may lead to misclassification in analyzes. Second, all the self-reported information may lead to misclassification bias or recall bias. Third, some patients with severe sequelae of the stroke did not participate in the survey, resulting in a selection bias. Fourth, although we have adjusted for several confounding variables, we have not been able to completely exclude confounding effects, especially those not measured, that could limit the validity of the findings. Fifth, we cannot determine a causal relationship with circulating 25(OH)D levels and stroke. Sixth, the relationship may be in the opposite direction, as stroke may have caused vitamin D deficiency through malnutrition or reduced sun exposure.

In conclusion, in this observational study, circulating 25(OH)D was related with stroke history in a non-linear, reverse J-shaped manner. The lowest stroke prevalence for circulating 25(OH)D concentration was ~60 nmol/L. Prospective observational studies and randomized controlled trials are needed to elucidate the link between these two entities, and the effect of vitamin D supplements on stroke prevention.

## Data availability statement

Publicly available datasets were analyzed in this study. This data can be found here: Centers for Disease Control and Prevention (CDC), National Center for Health Statistics (NCHS), National Health and Nutrition Examination Survey (NHANES), https://wwwn.cdc.gov/nchs/nhanes/Default.aspx.

## Ethics statement

The studies involving human participants were reviewed and approved by National Center for Health Statistics (NCHS) Research Ethics Review Board. The patients/participants provided their written informed consent to participate in this study.

## Author contributions

J-hP contributed to study design, analysis, and writing of the manuscript. S-lW and J-xM contributed to data collection and revision of the manuscript. LC and Y-fZ contributed to result interpretation. X-dW contributed to the guidance in formulating the design and execution of the study. All authors contributed to the article and approved the submitted version.
