# Supplementary material for: Serum 25-hydroxyvitamin D is associated with stroke history in a reverse J-shape
Source: Front Neurol. 2023 Jan 5;13:1050788. doi: 10.3389/fneur.2022.1050788 (PMC9851395; doi:10.3389/fneur.2022.1050788)
Supplement: Supplementary file 1 [file Table_1.DOCX]

Supplementary Material

## **Supplementary Tables**

Table S1 Distribution of continuous variables with missing data comparing complete data to results from pooling the datasets with imputed variables from multiple imputation

| Covariates | Missing data,n(%) | Complete data  (mean±sd) | Imputed datasets  (mean±sd) |
| --- | --- | --- | --- |
| BMI(kg/m2) | 586(1.45) | 29.18 ± 6.93 | 29.19 ± 6.92 |
| Cotinine(ng/mL) | 150(0.37) | 60.09 ± 129.28 | 61.68 ± 409.49 |
| TC(mmol/L) | 193(0.47) | 5.04 ± 1.10 | 5.04 ± 1.10 |
| HDLC(mmol/L) | 193(0.47) | 1.36 ± 0.41 | 1.36 ± 0.41 |
| Urine creatinine(umol/L) | 493(1.21) | 11240.07 ± 7229.44 | 11244.33± 7261.06 |

Abbreviations: BMI = body mass index; TC = total cholesterol; HDLC = high density lipoprotein cholesterol; sd = standard deviation.

Table S2 Adjusted association between 25(OH)D and stroke history in complete data and imputed datasets

| Variable | Complete data | Imputed datasets |
| --- | --- | --- |
|  | OR(95%CI)*P* | OR(95%CI)*P* |
| 25(OH)D quintile(nmol/L) |  |  |
| Q1(≤39.3) | 1.38(1.12,1.70)0.003 | 1.42(1.17,1.73)<0.001 |
| Q2(39.4 - 53.1) | 1.32(1.07,1.63) 0.010 | 1.31 (1.07,1.60)0.008 |
| Q3(53.2- 65.4) | ref | ref |
| Q4(65.5 -80.8) | 1.27(1.03,1.57) 0.025 | 1.22(0.99,1.49)0.052 |
| Q5(≥80.9) | 1.36(1.11, 1.66) 0.003 | 1.32(1.09,1.60)0.005 |

Notes: Adjusted for age, gender, ethnicity, marital status, education level, season of examination, BMI, cotinine, urine creatinine, TC, HDLC, hypertension, hypercholesterolemia, diabetes, asthma, emphysema, cardiac disease. Abbreviations: BMI = body mass index; TC = total cholesterol; HDLC = high density lipoprotein cholesterol; 25(OH)D = 25-hydroxyvitamin D; OR = odds ratio; CI = confidence interval.

Table S3 Adjusted odds ratio for the association of 25(OH)D and stroke history in prespecified subgroups

|  |  | 25(OH)D Q1 | 25(OH)D Q2 | 25(OH)D Q3 | 25(OH)D Q4 | 25(OH)D Q5 | *P* for interaction |
| --- | --- | --- | --- | --- | --- | --- | --- |
|  | n | OR(95%CI) | OR(95%CI) | OR(95%CI） | OR(95%CI) | OR(95%CI) |  |
| Gender |  |  |  |  |  |  | 0.065 |
| Male | 19967 | 1.11 (0.82, 1.50) | 1.41(1.06,1.87)^*^ | ref | 1.16 (0.88, 1.55) | 1.36(1.03, 1.80)^*^ |  |
| Female | 20665 | 1.67(1.24,2.25)^*^ | 1.23 (0.90, 1.70) | ref | 1.46(1.07, 1.99)^*^ | 1.43(1.06, 1.92)^*^ |  |
| Ethnicity |  |  |  |  |  |  | 0.363 |
| Mexican American | 7097 | 1.27 (0.73, 2.19) | 1.31 (0.78, 2.19) | ref | 0.94 (0.52, 1.69) | 1.49 (0.83, 2.68) |  |
| Other Hispanic | 3564 | 0.74 (0.31, 1.81) | 0.84 (0.38, 1.86) | ref | 1.18 (0.58, 2.42) | 0.73 (0.32, 1.66) |  |
| Non-Hispanic White | 17175 | 1.65(1.16,2.34)^*^ | 1.29 (0.92, 1.79) | ref | 1.24(0.92,1.68) | 1.38(1.04, 1.83)^*^ |  |
| Non-Hispanic Black | 8636 | 1.47(1.00,2.14)^*^ | 1.51(1.01,2.27)^*^ | ref | 1.74(1.12, 2.71)^*^ | 1.34 (0.86, 2.08) |  |
| Other Race | 4160 | 1.71 (0.73, 3.99) | 1.37 (0.57, 3.25) | ref | 1.20 (0.51, 2.82) | 1.88 (0.87, 4.03) |  |
| Education Level |  |  |  |  |  |  | 0.609 |
| Less than high school | 10244 | 1.34 (0.95, 1.89) | 1.44(1.03,2.02)^*^ | ref | 1.44(1.02, 2.03)^*^ | 1.29 (0.91, 1.83) |  |
| High school | 9372 | 1.33 (0.86, 2.04) | 1.29 (0.83, 1.98) | ref | 1.14 (0.74, 1.75) | 1.66(1.11, 2.46)^*^ |  |
| College or above | 20983 | 1.50(1.06,2.11)^*^ | 1.22 (0.86, 1.73) | ref | 1.20 (0.86, 1.68) | 1.18 (0.86, 1.63) |  |
| Marital Status |  |  |  |  |  |  | 0.708 |
| Living with partner | 24954 | 1.39(1.04,1.86)^*^ | 1.38(1.04,1.82)^*^ | ref | 1.19 (0.90, 1.56) | 1.31(1.01, 1.71)^*^ |  |
| Living without partner | 15658 | 1.33(0.97,1.82) | 1.23 (0.89, 1.70) | ref | 1.37(0.99, 1.89) | 1.39(1.02, 1.90)^*^ |  |
| Hypertension |  |  |  |  |  |  | 0.635 |
| Yes | 13613 | 1.44(1.13,1.84)^*^ | 1.32(1.03,1.69)^*^ | ref | 1.33(1.04, 1.70)^*^ | 1.39(1.10, 1.76)^*^ |  |
| No | 26885 | 1.19 (0.79, 1.80) | 1.22 (0.82,1.81) | ref | 1.13 (0.75, 1.69) | 1.25 (0.84,1.86) |  |
| hypercholesterolemia |  |  |  |  |  |  | 0.058 |
| Yes | 12763 | 1.71(1.27,2.30)^*^ | 1.46(1.08,1.97)^*^ | ref | 1.42(1.06, 1.90)^*^ | 1.80(1.36, 2.36)^*^ |  |
| No | 20873 | 1.15 (0.83, 1.60) | 1.16 (0.83, 1.61) | ref | 1.12 (0.80, 1.56) | 0.95 (0.68, 1.33) |  |
| Diabetes |  |  |  |  |  |  | 0.181 |
| Yes | 4910 | 1.37(0.94, 1.99) | 1.51(1.04,2.18)^*^ | ref | 1.28(0.87,1.88) | 1.36 (0.95, 1.96) |  |
| No | 35695 | 1.37(1.06,1.77)^*^ | 1.19 (0.92, 1.54) | ref | 1.26 (0.98, 1.62) | 1.35 (1.06,1.72)^*^ |  |
| Asthma |  |  |  |  |  |  | 0.519 |
| Yes | 5627 | 1.02 (0.64, 1.62) | 1.61(1.03,2.51)^*^ | ref | 1.40(0.88,2.21) | 1.47(0.96,2.26) |  |
| No | 34969 | 1.52(1.20,1.93)^*^ | 1.26(0.99,1.60) | ref | 1.25(0.99,1.58) | 1.34(1.07,1.69)^*^ |  |
| Chronic bronchitis |  |  |  |  |  |  | 0.380 |
| Yes | 2334 | 1.95(1.02,3.70)^*^ | 1.75 (0.89, 3.43) | ref | 1.90(0.97,3.73) | 2.10(1.13,3.91)^*^ |  |
| No | 38216 | 1.33(1.07,1.67)^*^ | 1.28(1.03,1.60)^*^ | ref | 1.21 (0.97,1.51) | 1.29 (1.04,1.60)^*^ |  |

Note: Adjusted for age, gender, ethnicity, marital status, education level, season of examination, BMI, cotinine, urine creatinine, TC, HDLC, hypertension, hypercholesterolemia, diabetes, asthma, emphysem and cardiac disease except the subgroup variable. Abbreviations: BMI = body mass index; TC = total cholesterol; HDLC = high density lipoprotein cholesterol; 25(OH)D = 25-hydroxyvitamin D; OR = odds ratio; CI = confidence interval; ref = reference. *P<0.05.
